# Low-field MRI’s Spark on Implant Safety: A Closer Look at Radiofrequency Heating

**DOI:** 10.1109/EMBC40787.2023.10340861

**Published:** 2023-07

**Authors:** Pia Sanpitak, Bhumi Bhusal, Jasmine Vu, Laleh Golestanirad

**Affiliations:** Department of Biomedical Engineering, Northwestern University, Evanston, IL, 60201 USA. She is now with the Department of Radiology, Northwestern University, Chicago, IL, 60611 USA; Department of Radiology, Northwestern University, Chicago, IL, 60611 USA.; Department of Biomedical Engineering, Northwestern University, Evanston, IL, 60201 USA and the Department of Radiology, Northwestern University, Chicago, IL, 60611 USA.; Department of Biomedical Engineering, Northwestern University, Evanston, IL, 60201 USA and the Department of Radiology, Northwestern University, Chicago, IL, 60611 USA.

## Abstract

Advances in low-field magnetic resonance imaging (MRI) are making imaging more accessible without significant losses in image quality. In addition to being more cost-effective and easier to place without as much needed infrastructure, it has been publicized that the lower field strengths make MRI safer for patients with implants. To test this claim, we conducted a total of 368 simulations with wires of various lengths and geometries in a gel phantom during radiofrequency (RF) exposure at 23 MHz and 63.6 MHz (corresponding to MRI at 0.55 T and 1.5 T). Our results showed that heating in the gel around wire tips could be higher in certain cases at 0.55 T. To examine the impact on real patients, we simulated two models of patients with deep brain stimulation (DBS) implants of different lengths. These simulations provide quantitative evidence that low-field MRI is not always safer, and this paper serves to illustrate some of the basic principles involved in RF heating of elongated implants in MRI environments.

## I. Introduction

In 2008, the World Health Organization reported that 90% of the world lacked access to magnetic resonance imaging (MRI) [1]. Although this percentage has been gradually decreasing, the COVID-19 pandemic has highlighted the need for accessible healthcare, including high-quality imaging. Low-field MRI is an attractive solution to these problems, especially in developing countries, due to its reduced cost and ease of siting. Technological improvements in electronics and magnetic resonance image construction now allow for lower field strengths to produce images with quality comparable to that of standard field strengths [2]. Additionally, the lower field strength reduces susceptibility and off-resonance artifacts, enabling imaging of previously difficult-to-image organs such as the lung [3]. For all of these reasons and more, there is growing interest among researchers and clinicians in using low and very-low field strength MRI systems.

Low-field MRI systems have been promoted as “implant-friendly”, a claim that is controversial. While the local specific absorption rate (SAR) to B_1_^+^ ratio is reduced at lower fields in the absence of implants, the presence of elongated implants can cause the “antenna effect” – the coupling of the leads with the electric field intensifies the SAR of radiofrequency (RF) energy in the tissue, which can lead to serious tissue damage [4]. Additionally, while scans of patients with implants have been safely performed in low-field MRI scanners under certain conditions (e.g., adult patients with cardiac implants) [5], there is little data on other cases involving implants, such with neuromodulation devices.

Here, we use a systematic series of simulations in 0.55 T and 1.5 T MRI scanners to examine how various implant-related factors impact heating in the tissue near the wire tip. We also take an initial look at the power deposition in the tissue surrounding implanted devices in different realistic patient groups. This paper seeks to provide additional evidence on whether low-field scanners truly reduce RF-heating as commonly assumed and highlights the need to consider a diverse range of patient populations.

## II. Methods

### A. MRI RF Coils

**Figure 1.**
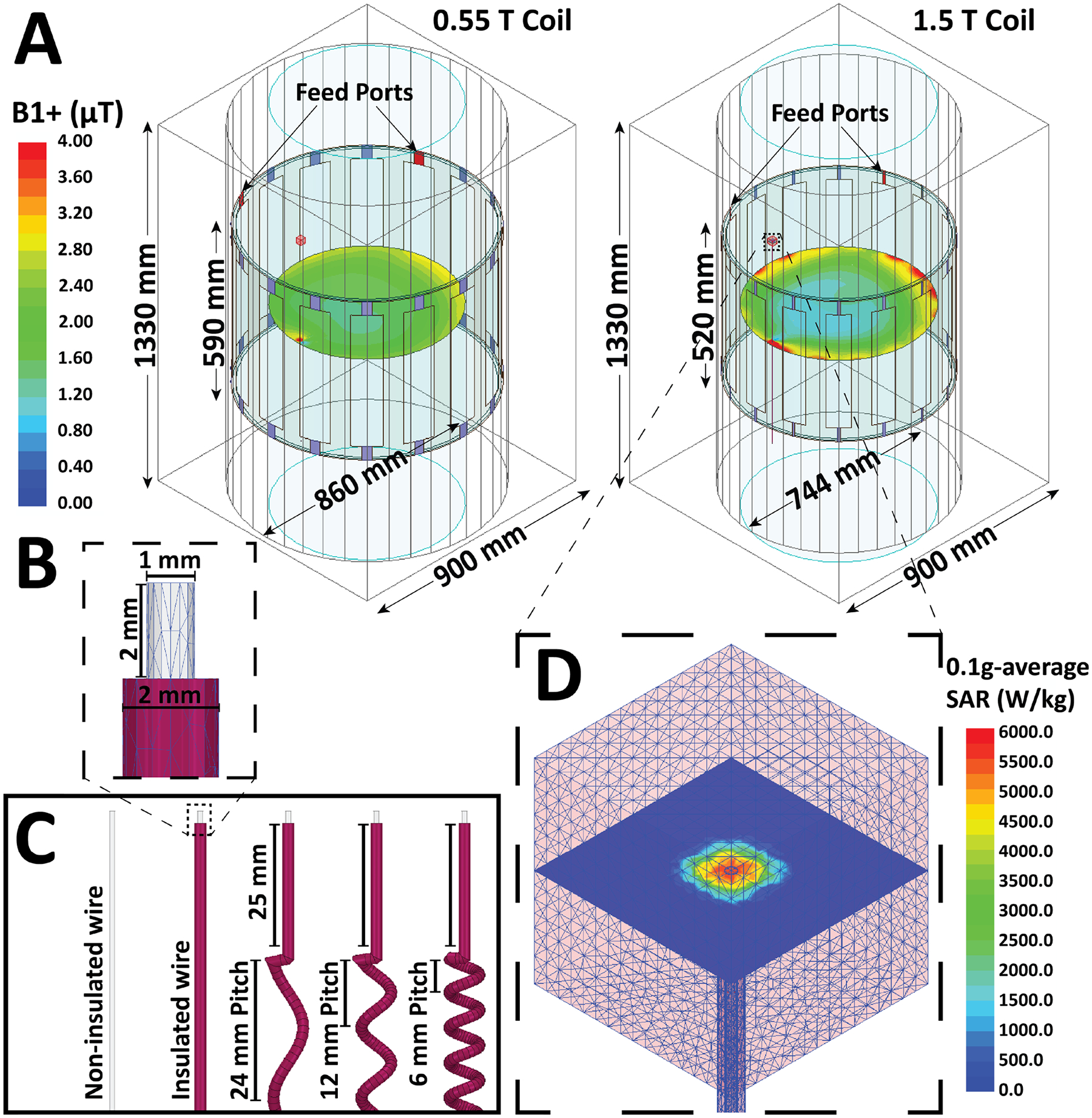
(A) Models of a 0.55 T and a 1.5 T birdcage coil loaded with a cylindrical phantom which contains a 75 cm straight insulated wire near the edge of the phantom. The input power of the coil is adjusted to generate B_1_^+^= 2 μT at the iso-center. (B) Dimensions of wire and tip. (C) Display of different wire pitches. (D) A close view of the high-mesh area surrounding the wire and tip, and the high gradient SAR field in the tissue near the tip.

Two 16-rung, circularly polarized, high-pass, RF body coils were implemented in ANSYS Electronics Desktop 2021 R1 (ANSYS Inc., Canonsburg, PA). The coils were tuned to operate at 23 MHz (corresponding to 0.55 T MRI) and 64 MHz (corresponding to 1.5 T MRI) using a combination of finite element method and circuit analysis [6,7]. The coil geometries were based on the Siemens 0.55 T Free.Max and Siemens 1.5 T Aera MRI scanners, excited through two ports on the top end ring separated by 90° in both phase and position (as shown in Fig. 1A). The coils were loaded with a cylindrical average tissue-mimicking phantom (diameter = 60 cm, length = 150 cm, σ = 0.5 S/m, ε_r_ = 64), and the input power of each coil was adjusted to generate a mean B_1_^+^ of 2 μT on a circular axial plane (diameter = 60 cm) passing through the iso-center of the coil. Care was taken to subtract the area of this plane that intersected with the lead to avoid any biased increase from the conductive object.

### B. Systematic Simulations with Wire Models

We created models of both insulated and uninsulated wires with varying pitches to examine the effect of the actual length of the wire (i.e., the electric length of entire wire when stretched out) and the apparent length of the wire (i.e., straight distance from the tip to the end of the wire) on RF heating different MRI environments. The wires were placed 15 mm away from the edge of the phantom. All wires were 1 mm in diameter and made of Platinum-Iridium (σ = 4 × 106 S/m, ε_r_ = 1). The insulated wires were embedded within a 2 mm diameter urethane insulation (σ = 0 S/m, ε_r_ = 3.5), and had a 2 mm exposed tip (Fig. 1B).

A total of 368 wire models were created, with apparent lengths that varied from 10 cm to 120 cm, spaced at intervals of 5 cm. For each apparent length, wires were modeled as straight and also with 3 different helical pitches, resulting in a total of four different actual lengths (as shown in Fig. 1C).

The accuracy of SAR calculations was improved by defining a high-mesh resolution cubic volume of 20 mm × 20 mm × 20 mm around the tip of each lead, with rms length = 1.5 mm. The maximum of the 0.1 g-average SAR (MaxSAR) was calculated and reported within this area (Figure 1D).

### C. Simulations with Realistic Deep Brain Stimulation (DBS) Lead Trajectories

**Figure 2.**
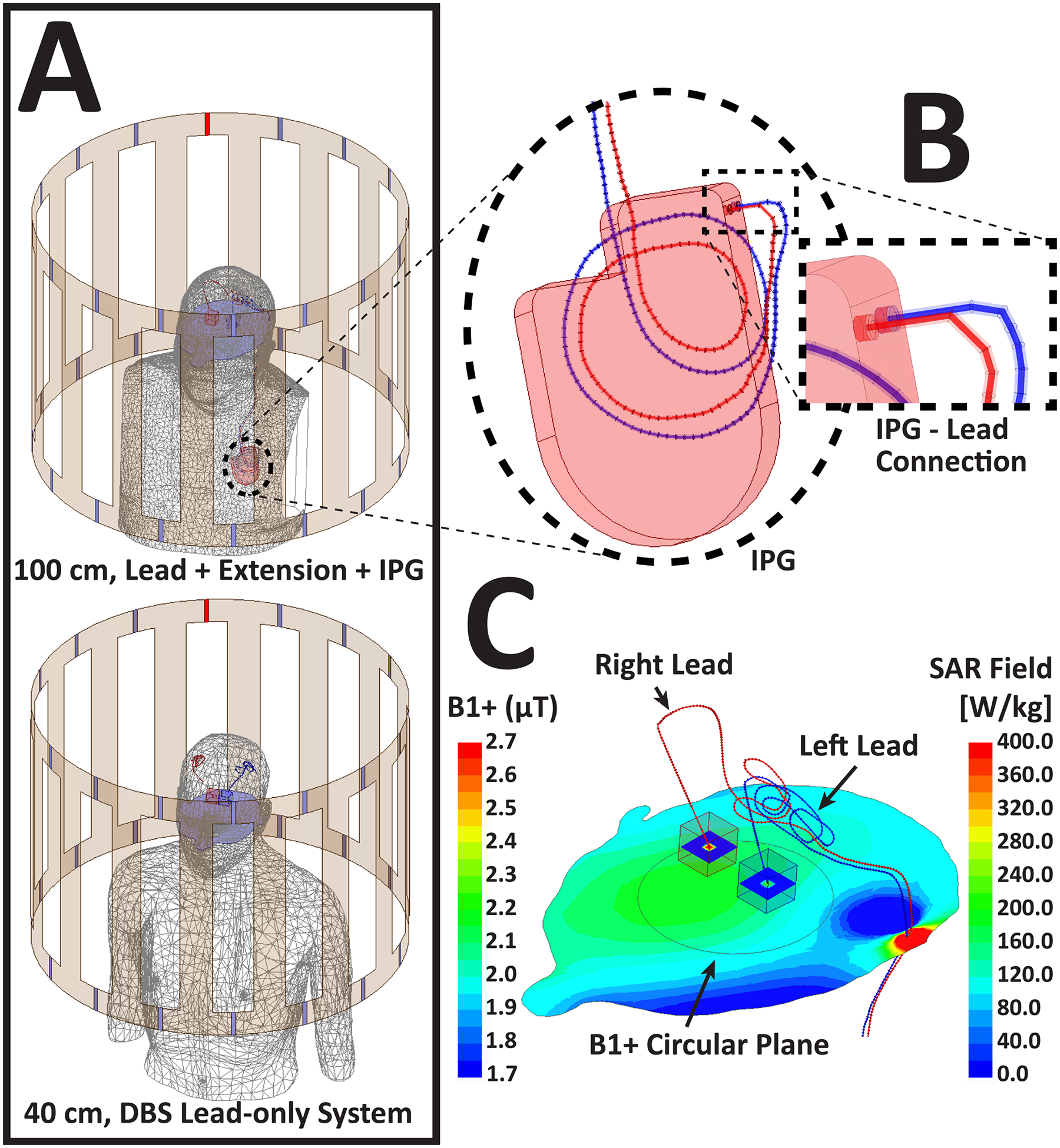
A) A DBS lead-only system with a 40 cm lead, and a full DBS system with a 40 cm lead connected to a 60 cm extension and a pulse generator (100 cm total length). B) Close view of the IPG-lead interface in the full system. C) Close up of the lead-only case the 1.5 T coil, showing the SAR fields near the tips of the leads and the B1+ field on an axial slice located in the center of the coil.

To simulate a more realistic scenario, two patient models with implanted deep brain stimulation (DBS) leads were placed inside the coils. Lead trajectories were based on the actual patient images obtained from post-operative computed tomography (CT) scans taken after DBS surgery [8]. The first model had a full DBS system, which consisted of a 40 cm lead, a 60 cm extension, and an implantable pulse generator (IPG), while the second model had a 40 cm lead-only system (Fig. 2A).

Trajectories of these models were extracted from the CT images of patients who underwent DBS implantation surgery in our institution [7]. DBS leads were modeled as Platinum-Iridium wires (σ = 4 × 10^6^ S/m, ε_r_ = 1) with 0.5 mm diameter embedded within a 1 mm diameter urethane insulation, with a 2 mm exposed tip. The proximal end of the lead was connected to the IPG with an insulation buffer in between (Fig. 2B). The MaxSAR was calculated in a similar manner to the previous section, and the input power of both coils were adjusted to have a mean B_1_^+^ of 2 μT on an axial circular plane (diameter = 4.8 cm) passing through iso-center (Fig. 2C).

## III. Results

### A. Wires with Straight Trajectories

**Figure 3:**
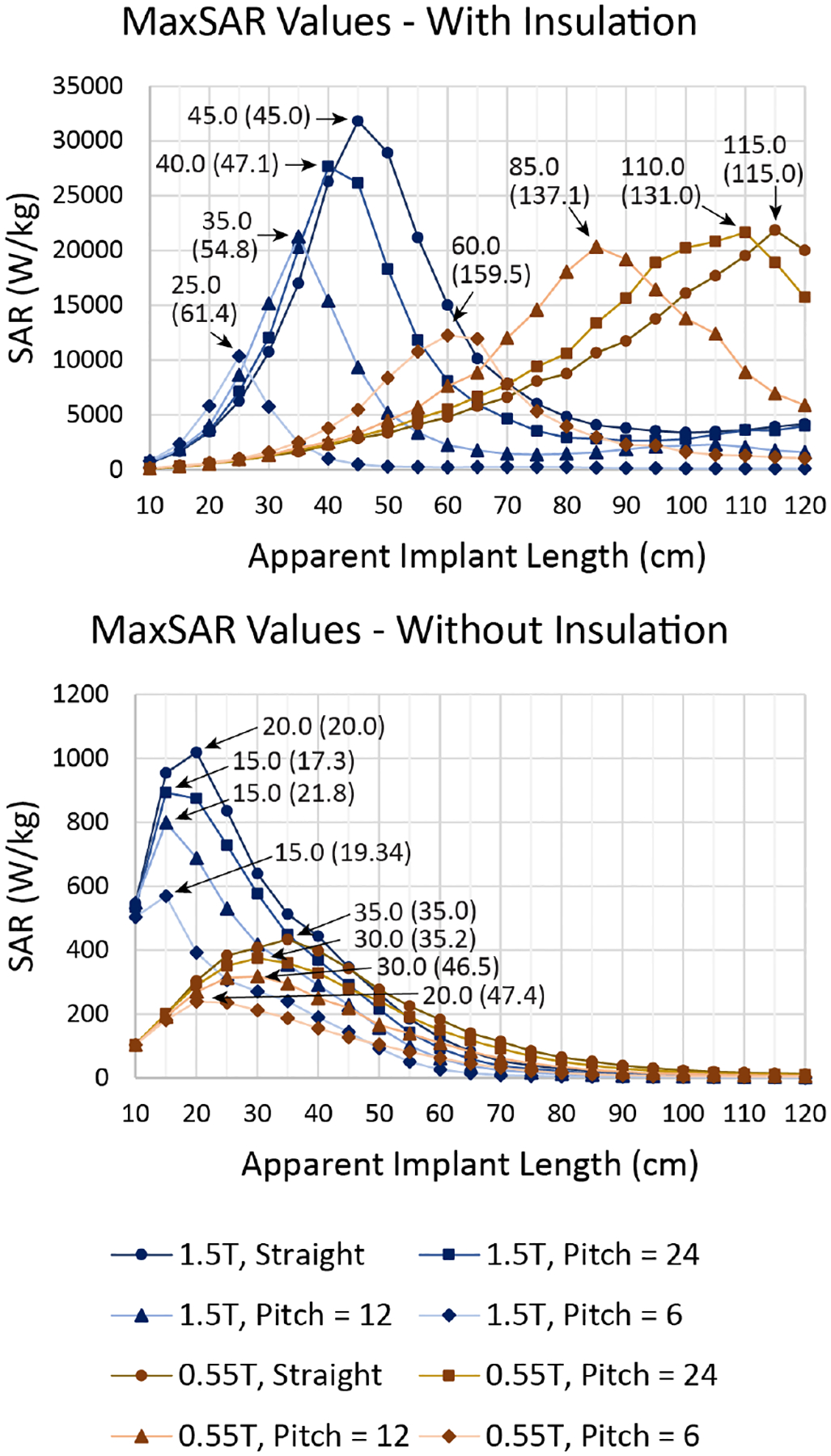
Maximum of 0.1g-averaged SAR (MaxSAR) around tips of insulated and uninsulated wires in the 1.5 T and 0.55 T RF coils. For all simulations, the input power of the coil was adjusted to generate a mean B1+ = 2 *μ*T on a central plane passing through the coil’s iso-center. The apparent and actual lengths (in parentheses) at which the peak occurred are denotated on the graphs.

For each wire geometry and each coil, there was a length at which the heating reached a peak before tapering off, for both insulated and bare leads. The heating was observed to be substantially higher for insulated leads compared to uninsulated leads. The MaxSAR was highest for a straight wire and decreased with the increase in helical pitches for both insulated (Fig. 3A) and uninsulated (Fig. 3B) leads.

For a straight, uninsulated wire, the peak MaxSAR was observed at around 20 cm in the 1.5 T coil and 35 cm in the 0.55 T coil. As the helical pitch of the coil increased, causing the actual length of the wire to become longer, the apparent length at which the peak occurred decreased and so did the height of the peak. There were some cases where the heating was lower with the 1.5 T coil than with the 0.55 T coil without insulation, but large differences were observed when the wires were insulated.

For a straight insulated wire, the peak MaxSAR was observed at around 45 cm in the 1.5 T Aera coil and 115 cm in the 0.55 T Free.Max coil. The presence of the insulation and the inductive coupling from the helix loops significantly altered the resonant length at which the peak MaxSAR was observed. In comparison, no such modulation was seen in uninsulated wires. Specifically, making a helical structure from the straight wire resulted in changes in the actual length at which resonance occurred. With a pitch = 6 mm, in which the implants had the tightest helical structure, the apparent length in which the peak MaxSAR occurred in the 1.5 T Aera coil was near 25 cm and the actual length was 61.4 cm. In the 0.55 T Free.Max coil, the peak was observed at around 60 cm, and the wire had an actual length of 159.5 cm.

### B. DBS Lead Models

**Figure 4:**
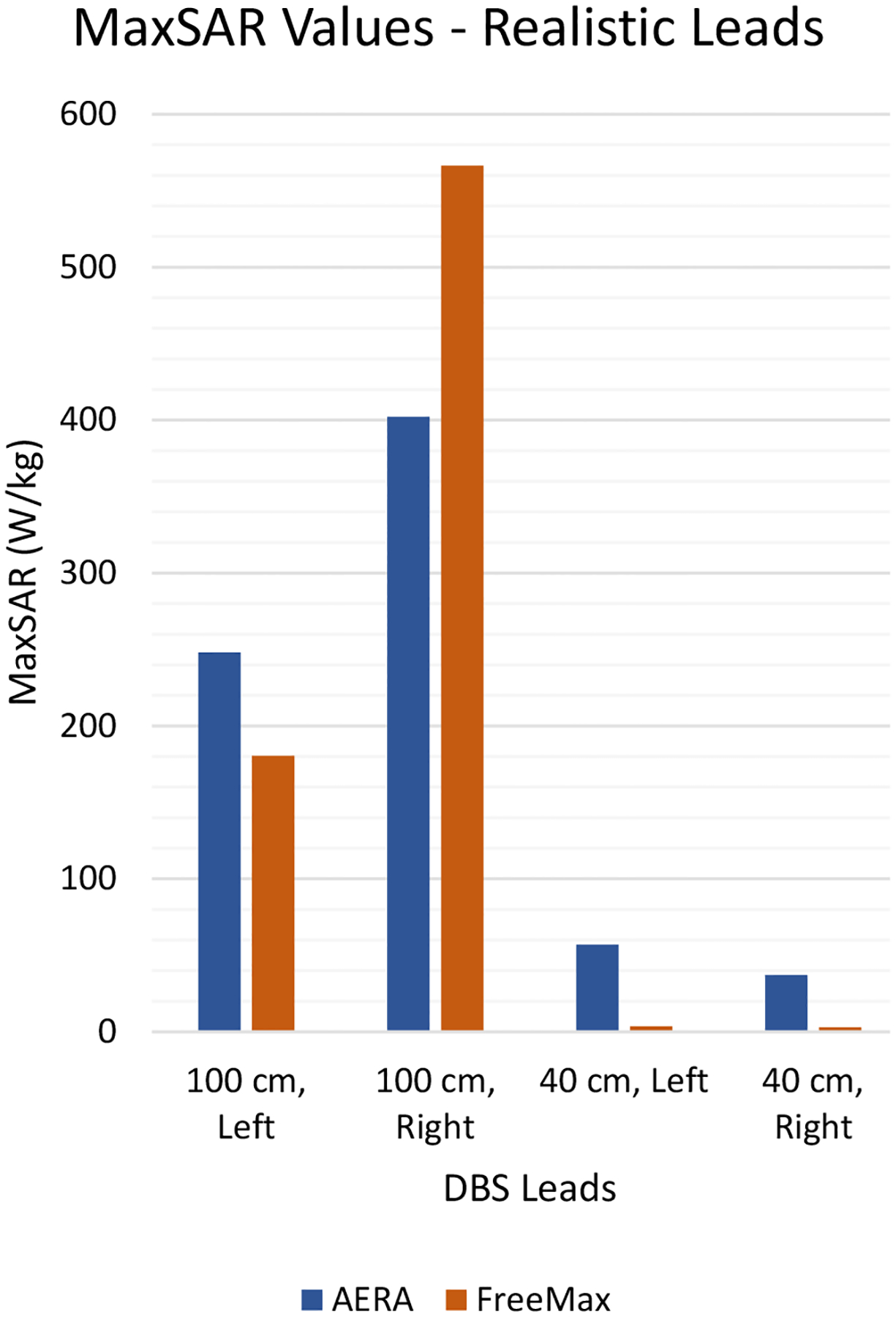
Results of Realistic DBS implant simulations.

Fig. 4 presents the results of simulations with DBS lead models. The MaxSAR at the tips of the lead-only system (40 cm lead) was significantly lower in the 0.55 T Free.Max coil compared to the 1.5 T Aera coil. The MaxSAR at the right lead tip was 37.1 W/kg at 1.5 T and 2.9 W/kg at 0.55 T. Similarly, the MaxSAR at the left lead tip was 57.1 W/kg at 1.5 T compared to 3.6 W/kg at 0.55 T. On the other hand, the MaxSAR values for the full system (lead + extension (total length 100 cm) + IPG) were more comparable between the two coils. Specifically, MaxSAR increased by 40.8% at the tip of the right lead in the full system at 0.55 T compared to 1.5 T.

## IV. Discussion

While MRI-induced RF heating for patients with electronic implants continues to be a significant issue, efforts to mitigate it have increased in recent years. Examples of these efforts include modifying the material and design of leads [9], introducing new MRI transmit technology to create a region of low electric field that aligns with the implanted lead’s trajectory on a patient-specific basis [10–15], and exploring the potential use of ultra-high-field [15,16] and vertical open-bore scanners that have different magnetic and electric field orientations [17–19]. Recently, low and ultra-low field MRI has also been touted as an additional option for safely imaging patients with conductive implants.

Our systematic simulations showed the clear impact of the resonance effect on the RF heating of elongated wires. The inductive coupling and increased length provided by the helical loops modified the length at which the resonance effect takes place. Our results showed that while changing the structure and length of uninsulated wires through the addition of helices had only a minimal impact on the resonant length, it had a significant impact on the insulated wires. These findings have important implications for real patients who typically have insulated leads, as our simplified simulations suggest that individuals with long leads may be more susceptible to increased RF heating at lower magnetic field strengths compared to higher fields.

This work has several limitations that should be acknowledged. One such limitation is the simplified geometry used in the simulations, as the human body is much more complex than a simple cylinder. Various factors, such as implant’s trajectory and the heterogeneity of surrounding tissue, can impact the heating of tissue near the tips of an elongated implant, and thus more work is needed to take these factors into account [7, 20, 21]. Additionally, while numerical simulations can reliably predict real-world scenarios when the details of the implant and MRI systems are properly represented [22], experimental measurements are ultimately needed to verify these predictions. To address these limitations, our future work includes conducting experimental measurements of RF response of various types of implants in low-field MRI scanners.

## Clinical Relevance—

This paper illustrates the physical concepts of resonance and inductive coupling in RF heating during MRI scanning with implants through systematic simulations and discusses the impact of these principles in practice.
